# Two Decades of Real-World Study in Newly Diagnosed Multiple Myeloma: Evolving Treatment and Outcomes in China with Reference to the United States

**DOI:** 10.3390/cancers18010053

**Published:** 2025-12-24

**Authors:** Jingyu Xu, Meng Shu, Hsingwen Chung, Jian Cui, Yuntong Liu, Wenqiang Yan, Qirui Bai, Ning Dai, Lingna Li, Jieqiong Zhou, Yating Li, Chenxing Du, Shuhui Deng, Weiwei Sui, Yan Xu, Hong Qiu, Lugui Qiu, Gang An

**Affiliations:** 1 State Key Laboratory of Experimental Hematology, National Clinical Research Center for Blood Diseases, Haihe Laboratory of Cell Ecosystem, Institute of Hematology & Blood Diseases Hospital, Chinese Academy of Medical Sciences & Peking Union Medical College, Tianjin 300020, China; xujingyu@ihcams.ac.cn (J.X.); cuijian@ihcams.ac.cn (J.C.); liuyuntong@ihcams.ac.cn (Y.L.); yanwenqiang@ihcams.ac.cn (W.Y.); qirui_bai@163.com (Q.B.); daining@ihcams.ac.cn (N.D.); lilingna@ihcams.ac.cn (L.L.); zhoujieqiong@ihcams.ac.cn (J.Z.); liyating@ihcams.ac.cn (Y.L.); duchenxing@ihcams.ac.cn (C.D.); dengshuhui@ihcams.ac.cn (S.D.); suiweiwei@ihcams.ac.cn (W.S.); xuyan1@ihcams.ac.cn (Y.X.); qiulg@ihcams.ac.cn (L.Q.); 2 Tianjin Institutes of Health Science, Tianjin 301600, China; 3 Global Epidemiology, Johnson & Johnson, Shanghai 200233, China; mshu3@its.jnj.com; 4 Global Epidemiology, Janssen Research and Development, LLC, Titusville, NJ 08560, USA; hchung17@its.jnj.com; 5 The Second Hospital of Shanxi Medical University, Taiyuan 030000, China

**Keywords:** multiple myeloma, real-world study, survival, treatment, autologous stem cell transplant

## Abstract

**Background**: The survival of newly diagnosed multiple myeloma (NDMM) has improved markedly worldwide with the introduction of proteasome inhibitors (PIs), immunomodulatory drugs (IMiDs), and anti-CD38 monoclonal antibodies. However, real-world progress among Chinese patients remains underexplored. This study evaluated 20-year survival trends in patients with NDMM treated in our institute and benchmarked them against outcomes from the Flatiron Health database in the United States. **Patients and methods**: Consecutive adults diagnosed with NDMM in our institute between 2003 and 2023 were retrospectively analyzed. U.S. patients were identified from the Flatiron Health database using similar inclusion criteria. Clinical characteristics, first-line regimens, and autologous stem cell transplantation (ASCT) rates were summarized. Overall survival (OS) and progression-free survival (PFS) were estimated by Kaplan–Meier methods. **Results**: Among 1622 Chinese and 12,582 US patients, median age was 57 vs. 68 years. The median PFS and OS of NDMM patients in our institute was 40.1 months and 99.6 months, respectively. Induction therapy in the NICHE cohort changed markedly from primarily chemo-based therapy to combined PIs + IMIDs-based treatment, whereas these treatments were used much earlier in Flatiron. Uptake of new therapies in China increased rapidly after their inclusion in national health insurance. ASCT utilization was higher overall in China (34.9% vs. 22.1%) but remained lower among patients >65 years (6.7% vs. 12.1%). **Conclusions**: Two decades of real-world data from a major Chinese myeloma center demonstrate substantial improvements in survival and modernization of NDMM treatment, while highlighting persistent disparities amongst older adults.

## Simple Summary

Multiple myeloma is a hematological malignancy whose treatment has improved greatly in recent years. However, long-term real-world evidence from China remains limited. In this study, we analyzed 20 years of data from one of the largest myeloma centers in China to examine changes in treatment patterns and patient survival. We found that survival has improved substantially alongside increased access to modern therapies and autologous stem cell transplantation. By placing these findings in the context of real-world data from the United States, this study highlights progress in myeloma care in China and points to remaining challenges, particularly among older patients.

## 1. Introduction

Multiple myeloma (MM) is the second most common hematological malignancy with increasing trends in incidence globally [1]. Worldwide, the age-standardized incidence rate of MM is 1.8 per 100,000 population, although considerable geographic variation exists; for example the age-standardized incidence rate is 4.8 per 100,000 population in the United States (U.S.), versus 1.2 per 100,000 population in China [2], a difference that is largely attributable to population age structure, as MM incidence increases steeply with age. Data from the Global Burden of Disease 2021 study show that MM incidence and mortality in China have increased steadily since 1990, with cases projected to rise further for the next 25 years as the population ages [3,4].

Over the past two decades, the development of proteasome inhibitors (PIs), immunomodulatory drugs (IMiDs), and anti-CD38 monoclonal antibodies has revolutionized the management of newly diagnosed multiple myeloma (NDMM), extending the survival of patients with NDMM significantly both in clinical trials and real-world practice [5,6,7,8,9]. Despite these advances, geographic and economic disparities continue to influence patient outcomes and access to novel therapies and transplantation resources remains heterogeneous across regions [10,11]. In China, access to innovative anti-myeloma agents historically lagged behind high-income countries due to a documented “drug-lag” and limited reimbursement coverage [12], but the introduction of the National Reimbursement Drug List (NRDL) and accelerated drug approval policies in recent years have markedly improved accessibility [13,14].

The Institute of Hematology & Blood Diseases Hospital, Chinese Academy of Medical Sciences and Peking Union Medical College is one of the largest referral centers for MM in China, which has treated thousands of patients with MM since the early 2000s. With two decades of continuously collected real-world data, our institute provides a unique opportunity to examine long-term trends in survival and treatment evolution within a consistent clinical environment.

Although some studies in China have shown the substantial improvement in MM outcomes [8,15], few have contextualized these trends within the global real-world landscape. To provide a broader context to the Chinese setting, data from the Flatiron Health database in the U.S., which is widely regarded as a benchmark for mature and high-resource healthcare systems, were used as an international reference. This study aimed to characterize the evolution of treatment and outcomes in patients with NDMM from 2003 to 2023 in our institute and to explore the clinical and therapeutic factors that have driven this progress, thereby identifying areas for further optimization and alignment with global standards of myeloma care.

## 2. Patients and Methods

### 2.1. Study Design and Data Sources

This retrospective real-world cohort study evaluated longitudinal outcomes of patients with NDMM treated in our institute between January 2003 and June 2023 and benchmarked these results against contemporaneous data from the Flatiron database.

Data for the Chinese cohort were obtained from the National Institute of Hematology and Clinical Excellence (NICHE) registry, a hospital-based real-world database established and maintained by our institute (ClinicalTrials.gov identifier NCT04645199). The NICHE registry systematically integrates electronic medical records (EMRs) with structured telephone follow-ups, focusing on the patient’s current treatment regimen, therapeutic efficacy, disease progression and the time of progression, as well as the cause and date of death.

The Flatiron Health cohort, representing more than 280 community and academic oncology sites in the U.S., was selected to provide a national real-world benchmark. Multiple analyses have used Flatiron’s EMR-derived cohort to characterize treatment patterns and outcomes, supporting its use as an international real-world benchmark [16,17]. De-identified EMR-derived data from 2003 to 2023 were extracted using the same inclusion criteria applied to the NICHE cohort, ensuring consistency in variable definitions. Mortality data are derived from a consensus variable using three data sources (the EMR, Social Security Death Index, and obituary data).

Because of limited patient numbers and incomplete follow-up in the early years of data collection, analyses of age-standardized death rate, progression-free survival (PFS), and overall survival (OS) were restricted to patients diagnosed from 2008 onward. This cutoff coincides with the full implementation of EMRs and standardized data capture at both NICHE and Flatiron, ensuring reliability and comparability of survival estimates. Cases diagnosed before 2008 were included only in descriptive baseline summaries.

Eligible patients were required to be at least 18 years old at the time of the initial diagnosis of MM. The index date was defined as the date of first MM diagnosis, with the baseline period set as 12 months prior to the index date. Patients in both databases were followed up until the earliest occurrence of death, the last available medical record, or the study end date (30 June 2023). Patients were excluded from the analysis if there was evidence of prior treatment for MM (relapsed or refractory MM) during the baseline period, or who were untreated or without consecutive treatments, which was defined as ≥4 cycles of induction therapy.

### 2.2. Variables and Endpoints

Demographic and clinical information including year of diagnosis, International Staging System (ISS), isotype, cytogenic information, treatment patterns including types of induction treatment, ASCT, and maintenance regimens were recorded in both databases.

PFS was defined as the time from the index date until either the date of disease progression, death, the date of last follow-up, or the end of the study period, whichever occurred first. OS was the period of time from the index date until death from any cause, the date of last follow-up, or the end of the study period, whichever occurred first.

### 2.3. Statistical Analysis

Demographic and clinical categorical characteristics were described by frequencies and percentages, and continuous variables with means and standard deviation (SD). The Exact method was used to calculate 95% CIs. The proportion of missing data was described. Survival outcomes were estimated by the Kaplan–Meier method and compared using the log-rank test. Cox proportional hazards regression was applied to identify independent predictors of PFS and OS. Proportionality of hazards assumption was assessed by Log-minus Log Survival Plot, and multivariable stratified Cox model was conducted, stratifying by variables that do not meet the proportional hazards assumption and adjusted by clinically relevant variables such as ISS stage, cytogenetic risk, induction regimen, ASCT and maintenance therapy. Estimates of age-standardized mortality used Chinese national census data (2020) and U.S. National Vital Statistics Reports (2021). The analysis was performed using SAS 9.4 (SAS Institute, Cary, NC, USA) and graphs were created in R studio 4.1.2.

## 3. Results

### 3.1. Survival Improvement Among Chinese Patients with NDMM

Among 3357 patients in NICHE with at least one diagnosis of MM during the study period, 1622 patients who met the criteria were included in the analysis (Figure S1A).

**Figure 1:**
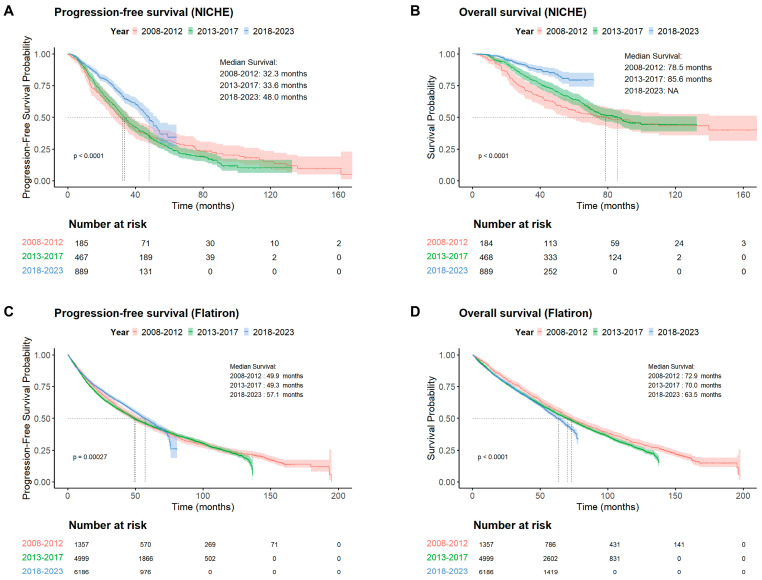
Progression-free survival and overall survival of all patients in the NICHE (**A**,**B**) and Flatiron (**C**,**D**) cohorts. NICHE: National Longitudinal Cohort of Hematological Disease in China, PFS: progression-free survival; OS: overall survival.

The median follow-up period was 35.6 months (IQR 22.29, 59.08) for NICHE and the age-standardized death rate in the NICHE cohort was 16.48%, vs. 0.57% in the general population of China (Table S1). The median PFS in all patients over the study period was 40.1 months (95% CI: 37.2–42.8) and median OS was 99.6 months (95% CI: 88.3–NR). Over the past two decades, patients with NDMM treated in China have achieved continuous and substantial improvements in PFS and OS (Figure 1). Median PFS increased from 32.3 months (95% CI: 26.7–38.9) in 2008–2012, to 33.6 months (95% CI: 30.7–36.7) in 2013–2017, and to 48.0 months (95% CI: 44.6–52.5) in 2018–2023 (Figure 1A). Corresponding median OS values were 78.5 months (95% CI: 56.2–NR), 85.6 months (95% CI: 72.4–NR), and not reached (95% CI: NR–NR) (Figure 1B). The 5-year PFS improved from 31.28% during 2008–2012 to 34.51% in 2018–2023, while OS increased from 55.38% to 79.41%.

The most pronounced improvement was observed after 2018, a period that also marked the increasing availability of novel agents and more intensive combination regimens in routine clinical use. These findings confirm a sustained and substantial survival improvement within this high-volume Chinese center.

### 3.2. Benchmarking Against US Real-World Outcomes

To contextualize these gains, outcomes at NICHE cohort were benchmarked against contemporaneous data from the Flatiron Health database in the U.S. In Flatiron, 17,529 patients were identified with at least one diagnosis of MM during the same period, of whom 12,582 met the inclusion criteria. (Figure S1B).

The median follow-up period was 38.83 months (17.80, 69.53) and the age-standardized death rate in the Flatiron cohort was 21.99%, vs. 0.76% in the general US population (Table S1).

In Flatiron, median PFS was 53.8 months (95% CI: 51.6–55.7) and median OS was 68.3 months (95% CI: 66.8–70.5) overall, with notable improvements in PFS but relatively stable OS from 2008–2023 (Figure 1C,D).

Although PFS at our center remained shorter than that observed in the U.S. Flatiron cohort, OS in the NICHE cohort showed substantial improvement in recent years. These findings suggest continued progress in the survival of Chinese NDMM patients and a gradual narrowing of historical outcome differences. To better understand the drivers of this improvement, we next explored potential contributing factors that may explain the rapid progress observed in China and provide insight for optimizing clinical practice.

### 3.3. Exploring Potential Reasons for Improved Survival

#### 3.3.1. Baseline Characteristics and Population Profile

The median age of patients was 57 years (range 25–83) in the NICHE cohort and 68 years (range 19–85) in the Flatiron cohort, with 18.37% of NICHE patients versus 60.01% of Flatiron patients aged >65 years. Males outnumbered females in both cohorts. At diagnosis, 41.77% of patients in the NICHE cohort were of ISS stage III versus 32.95% in the Flatiron cohort. IgG was the most frequent subtype in both cohorts, although less common in NICHE (48.36% vs. 59.08%), whereas IgD myeloma was observed more frequently (5.90% vs. 0.40%). High-risk cytogenetics were identified in 24.10% and 25.08%, respectively, defined by the presence of del(17p), t(4;14), or t(14;16) (Table 1).

These data suggest a younger, transplant-eligible population in China but otherwise broadly comparable disease biology between cohorts.

#### 3.3.2. Age Subgroup Analysis

Given that the age distribution differed substantially between cohorts, we next analyzed survival trends among patients aged ≤65 years old in the two cohorts. Demographic and disease characteristics of patients aged ≤65 years were comparable between the NICHE and Flatiron cohorts with respect to age and sex distribution. The distribution of ISS stages at diagnosis, as well as patterns of immunoglobulin isotype and cytogenetic risk, mirrored those observed in the overall study population (Table 2).

**Figure 2:**
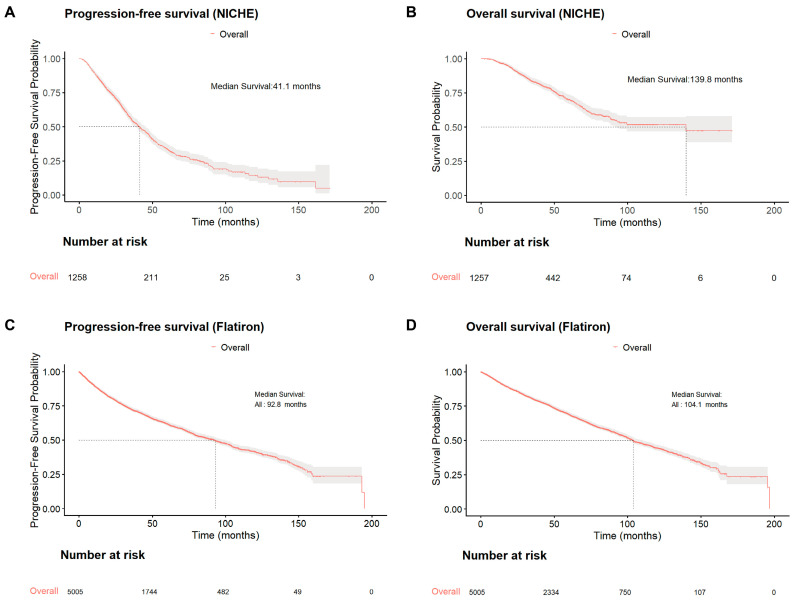
Progression-free survival and overall survival of patients ≤ 65-years of age in the NICHE (**A**,**B**) and Flatiron (**C**,**D**) cohorts. NICHE: National Longitudinal Cohort of Hematological Disease in China, OS: overall survival, PFS: progression-free survival.

Among Chinese patients aged ≤65 years old (Figure 2A,B), median PFS was 41.1 months (95% CI: 37.5–45.0) and median OS 139.8 months (95% CI: 92.6–NR). In the Flatiron reference dataset (Figure 2C,D), younger patients showed longer survival, with median PFS 92.8 months (95% CI: 86.4–99.7) and OS 104.1 months (95% CI: 100.3–110.2).

The prognosis of younger patients in both cohorts becomes better than that of the overall patients. The consistent upward trajectory in both PFS and OS highlights a rapid narrowing of the gap and ongoing convergence with international outcomes, likely reflecting parallel advances in treatment access.

#### 3.3.3. Evolution of Induction Therapy

**Figure 3:**
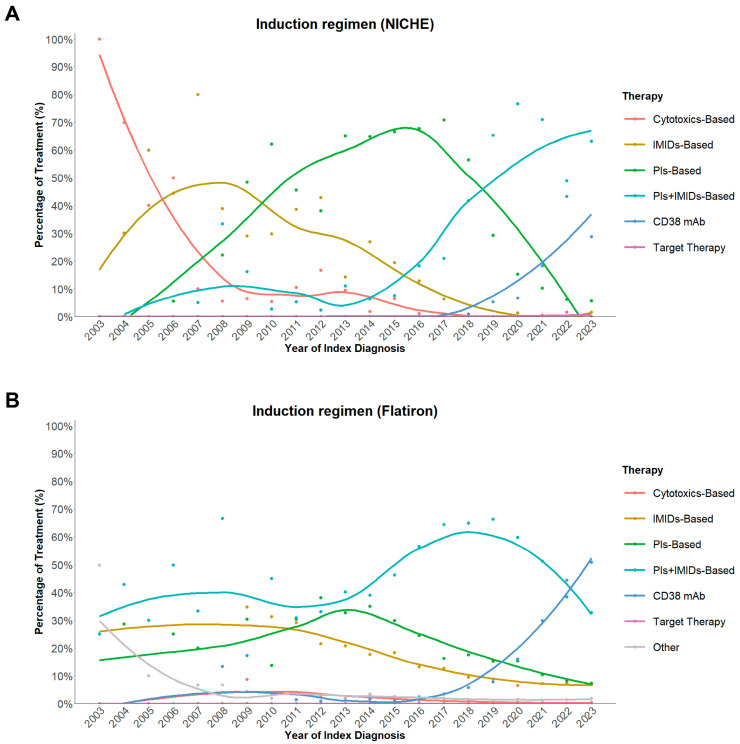
Trends in induction regimens over time in the NICHE (**A**) and Flatiron (**B**) cohorts *. IMID: immunomodulatory drug, mAb: monoclonal antibodies, NICHE: National Longitudinal Cohort of Hematological Disease in China, PI: proteasome inhibitor, Other: Rituximab ± Glucocorticoid. PI-based: regimens containing PI but without IMID or CD38 mAb; IMID-based: regimens containing IMID without PI or CD38 mAb; PI + IMID–based: regimens that included both PI and IMID without CD38 mAb; CD38 mAb–based: any regimen that included a CD38 monoclonal antibody, irrespective of other components. * Locally Estimated Scatterplot Smoothing was used for curves.

**Figure 4:**
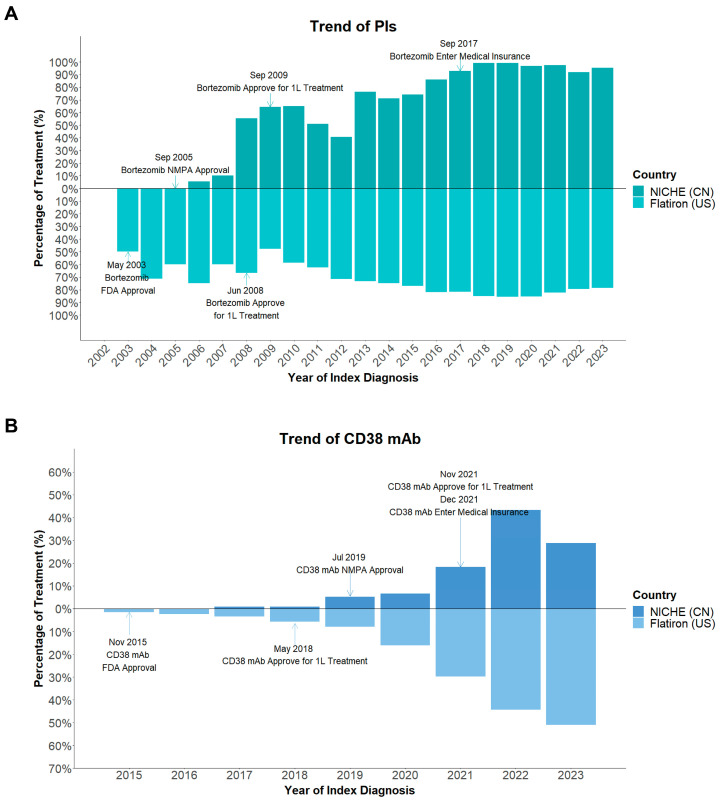
Bortezomib (**A**) and CD38 mAb (**B**) approval timeline in China and the U.S. 1L: first line, CN: China, FDA: US Food and Drug Administration, NICHE: National Longitudinal Cohort of Hematological Disease in China, NMPA: National Medical Products Administration, US: United States.

The evolution of frontline treatment patterns (Figure 3A) closely followed the timeline of drug approval and reimbursement in China (Figure 4). From 2003 to 2009, cytotoxics- and IMiD-based regimens predominated. The proportion of PI-based regimens began to rise gradually after bortezomib was approved for first-line use in 2009, and IMiD-based regimens alone started to decline. Although international guidelines had already recommended PI + IMiD–based combinations as the preferred standard of care, their use in China increased only slowly due to the high out-of-pocket cost of novel agents. Following inclusion of bortezomib in the NRDL in 2017, adoption of combined PI + IMiD regimens accelerated rapidly and became predominant thereafter. This policy milestone coincided with increasing awareness of evidence supporting triplet therapy, particularly after publication of the SWOG S0777 trial in 2017, which demonstrated superior PFS and OS with bortezomib-lenalidomide-dexamethasone (VRd) compared with lenalidomide-dexamethasone (Rd) in newly diagnosed patients without intent for immediate ASCT [18]. After daratumumab received National Medical Products Administration (NMPA) approval in 2019 and NRDL reimbursement for use in 2021, anti-CD38–containing regimens also increased steadily. By 2023, around 60% of patients with NDMM received induction with a PI + IMID-based regimen, and about 30% received anti-CD38 monoclonal antibodies as part of their treatment.

By contrast, IMiD- and PI-based regimens had been widely used in the U.S. much earlier, and anti-CD38 therapy expanded rapidly after FDA approval (Figure 3B and Figure 4). But the whole treatment evolving trend in our institute was similar with that in the U.S., with fewer cytotoxics- and IMiD-based regimens and more PI + IMID-based and anti-CD38 utilization.

Overall, these data demonstrate that while drug adoption in China initially lagged behind global practice, policy reform and expanded reimbursement have markedly accelerated access to novel therapies in recent years, leading to a progressive alignment of Chinese clinical practice with international therapeutic standards.

#### 3.3.4. ASCT Utilization and Trends

**Figure 5:**
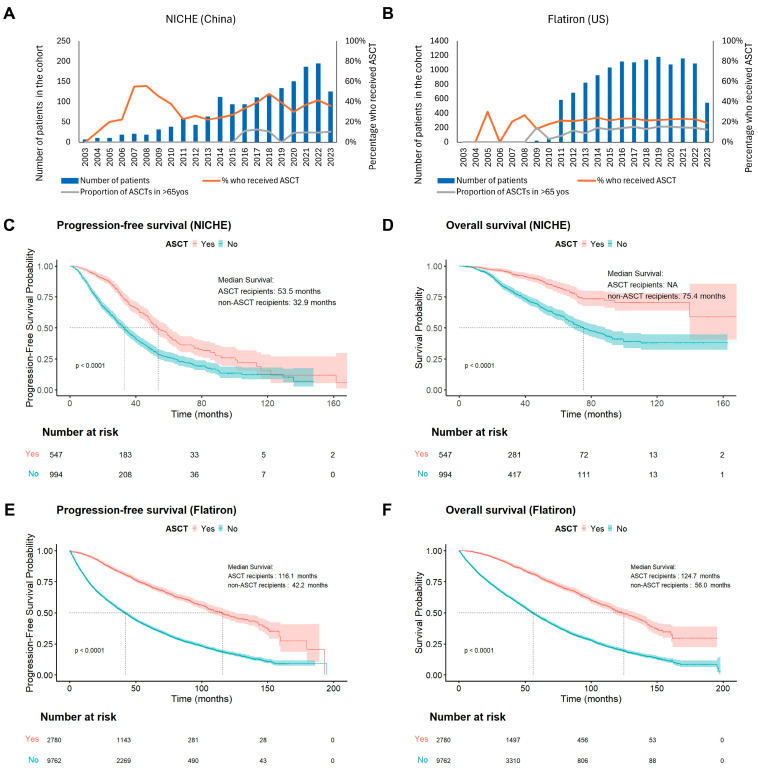
Trends in the use of ASCT (**A**,**B**) and survival of patients who underwent ASCT in the NICHE (**C**,**D**) and Flatiron (**E**,**F**) cohorts. ASCT: autologous stem cell transplant, NICHE: National Longitudinal Cohort of Hematological Disease in China, US: United States.

In the NICHE cohort, 566 (34.9%) patients with NDMM underwent first-line ASCT (Figure 5A). Over the past decade, the proportion of patients receiving ASCT has steadily increased, ranging from 22% to 48% annually. Among transplanted patients, 96.5% (*n* = 546) were aged ≤65 years, corresponding to an overall ASCT rate of 41.2% within this subgroup. Although ASCT remained uncommon among older patients, 20 individuals (6.7%) aged >65 years received ASCT during the study period, and their proportion among all transplants rose from 3% in 2016 to 7% in 2023, reflecting a gradual expansion of eligibility criteria and improved access to transplant care.

In the Flatiron reference dataset, 22.1% (*n* = 2785) of NDMM patients received first-line ASCT between 2003 and 2023 (Figure 5B). The annual ASCT rate remained relatively stable at 18–24% after 2010, with increasing use among patients aged >65 years over time.

Consistent with prior real-world evidence, ASCT recipients in the NICHE cohort achieved substantially longer survival than non-transplanted patients (Figure 5C,D). Among patients, median PFS reached 53.5 months, and median OS was NR, compared with 32.9 months and 75.4 months, respectively, in those who did not undergo ASCT. Similar trends were observed in the patients ≤ 65 years of age (Figure S2A,B). This pattern was also in line with findings from Flatiron (Figure 5E,F and Figure S2C,D), underscoring ASCT’s enduring value in improving outcomes for eligible patients with NDMM in the modern treatment era.

#### 3.3.5. Maintenance Trends

We analyzed patterns of maintenance therapy use across the NICHE and Flatiron cohorts. In the NICHE cohort, the use of maintenance therapy increased steadily over time. From 2003 to 2007, 25.0% of patients received maintenance therapy, rising to 54.0% by 2008–2012, 64.0% by 2013–2017, and 78.8% by 2018–2023. (Figure S3A–D). However, maintenance therapy data were incomplete in the earlier years, particularly before 2013, and this may influence the interpretation of survival outcomes.

The use of maintenance therapy in the Flatiron cohort was also observed to increase, although the rates remained lower than those in the NICHE cohort. The proportion of patients receiving maintenance therapy ranged from 27.5% in 2003–2007 to 30.4% in 2008–2012, reaching 35.8% in 2013–2017, and 36.4% in 2018–2023 (Figure S3E–H). The differences in maintenance therapy rates between NICHE and Flatiron may reflect disparities in treatment protocols, reimbursement policies, and overall access to care in these two cohorts.

### 3.4. Prognostic Factors for Survival

Cox proportional hazards regression was performed to identify prognostic factors for survival in both cohorts (Table 3). At diagnosis, advanced ISS stage and high-risk cytogenetics were significantly associated with poorer outcomes. In treatment-related variables, multivariate analysis in the NICHE cohort confirmed that ASCT, and PI + IMiD–based or CD38 antibody–based induction were independent predictors of improved PFS and OS. Similar findings were observed in the Flatiron dataset, where ASCT remained strong predictors associated with prolonged survival. and the use of PI + IMiD and anti-CD38 therapy was significantly associated with PFS.

Age was an independent prognostic factor in the US cohort but not in the Chinese cohort, likely reflecting the younger population structure at China.

## 4. Discussion

This real-world study provides a comprehensive and longitudinal overview of the evolving treatment landscape and improved survival among patients with NDMM in one of the largest MM centers in China over the past two decades. We used the Flatiron Health database as a contextual benchmark to place these improvements within a global real-world setting. The results demonstrated a narrowing of historical outcome gaps and continuous advances in treatment strategies and clinical practice in our institute.

Our results align with several other published datasets and population-based studies that showed improved outcomes for patients with MM over time. Two national medical centers in China reported improved outcomes over 15 years as novel agents became standard [8,19], and a multicenter study similarly documented modernization of first-line treatment patterns and longer survival in the contemporary era [15]. Taken together, these data suggest that our single-center experience reflects the broader progress in MM care across China rather than an isolated outlier. Other studies from Norway [20], Spain [9] and the U.S. [7,21], also documented significant improvement in the last few years.

Over the study period, both PFS and OS improved steadily in NICHE patients. After 2018, a more pronounced upward inflection was observed, which temporally aligned with the reimbursement of PIs in 2017, and the use of combination regimens incorporating both PIs and IMiDs rose sharply. Subsequent approval of daratumumab in 2019 and its reimbursement for first line use in 2021 further expanded the use of anti-CD38–containing regimens (Figure 3 and Figure 4). These policy milestones, together with growing clinical experience, effectively accelerated the adoption of internationally recommended triplet and quadruplet regimens in routine Chinese practice. At the same time, the landmark clinical trial SWOG S0777 results influenced broader adoption of combination regimens [18]. The direction and magnitude of survival gains observed in our institute are consistent with other Chinese real-world cohorts [8,15,19]. ASCT remains an important treatment for eligible patients with MM and is associated with better prognosis as verified in many trials and real-world studies [21,22,23]. In our institute, transplanted patients also achieved markedly longer PFS and OS than patients who did not undergo ASCT. Previous reports have shown that ASCT can also benefit elderly patients [24,25]. In our analysis, only 4% of ASCT procedures in NICHE cohort involved patients over 65 years old, compared to 33% in the US (Figure 5). This divergence likely reflects multifactorial barriers—including clinician and patient perceptions of frailty/tolerability, transplantation center availability, and affordability/information gaps—that have been described as common obstacles to ASCT access [26,27,28]. These observations underscore an opportunity in China to expand fitness-based assessment, strengthen support, and improve referral pathways and financial access so that eligible older adults can realize the proven benefits of ASCT.

The survival data of U.S. is consistent with previous analyses of MM patients from Flatiron database [7,29]. Initial benchmarking suggested that OS among NICHE patients appeared longer than in the U.S. Flatiron population. Factors contributing to this observation are first, patients in NICHE cohort were substantially younger than in the US, as reported previously at our institution [30,31,32], with a higher proportion of transplant-eligible individuals. By contrast, the Flatiron cohort reflects an older, community-based U.S. population. Age is one of the strongest predictors of MM survival, and this demographic difference alone is expected to contribute to longer OS in China. Furthermore, as the largest hematology referral and treatment center in China, our institute has implemented standardized diagnostic and therapeutic protocols, which likely contribute to good outcomes in this cohort. The Flatiron database aggregates data from over 280 U.S. oncology centers with heterogeneous practice settings, potentially leading to greater variability in outcomes across sites. These center-level advantages may inflate survival estimates in comparison with population-based datasets.

Although Chinese patients achieved longer OS than those in the U.S. Flatiron cohort, their PFS remained shorter. This pattern of shorter PFS but longer OS in the NICHE cohort can be understood as the combined effect of (i) delayed widespread access to novel agents before 2017, resulting in fewer deep early responses and earlier progression; (ii) greater use of ASCT and higher maintenance intensity in China, prolonging survival after progression; (iii) younger patient age and stronger functional status at baseline; and (iv) the availability of broader treatment options after progression, as our institute is the largest hematology hospital in China, providing patients with access to more advanced therapeutic strategies, including clinical trial participation. These findings highlight the need to further improve early treatment efficacy and relapse detection in China while maintaining robust long-term management strategies.

Policy reforms have also played a pivotal role in driving these advances. Inclusion of key anti-myeloma agents in the NRDL substantially reduced costs and expanded access (Figure 4), while accelerated regulatory pathways shortened the approval lag between global and domestic markets. These systemic measures, together with ongoing efforts to enhance clinical capacity and regional equity, are expected to sustain long-term improvement in MM survival across China.

Several limitations should be acknowledged. First, selection bias is possible, as NICHE represents a single high-volume national referral center, which may result in higher survival estimates compared to population-based statistics. Additionally, we benchmarked treatment patterns and outcomes against the U.S. Flatiron database, which captures a broader community-based population. Second, patient numbers in both cohorts were relatively small before 2011, likely due to incomplete EMR documentation, and differences in data capture and follow-up methodology may introduce measurement bias. Third, key variables such as R-ISS stage, ECOG performance status, depth of response, and cytogenetic testing were missing or incomplete, particularly in early years, limiting our ability to fully adjust for fitness and disease biology. Lastly, differences in socioeconomic and healthcare systems between the two cohorts may have influenced treatment access and outcomes, with survival improvements in China closely tied to policy-driven insurance reforms. These limitations highlight that our analyses are descriptive and do not imply direct equivalence between China and the U.S. Further research involving multicenter, nationally representative cohorts is needed to validate these findings.

## 5. Conclusions

In summary, this two-decade real-world analysis from our institute demonstrates remarkable survival improvement among Chinese patients with NDMM paralleling the accelerated adoption of novel agents and increasing utilization of ASCT. Although the approval and reimbursement of novel therapies in China historically lagged behind Western countries, their rapid clinical integration at high-volume centers has enabled outcomes that now approach those observed in mature international cohorts. These findings highlight that systematic investment in access, infrastructure, and evidence-based practice can accelerate progress in MM management.

## Figures and Tables

**Table 1:** Demographic and clinical features of the NICHE (China) and Flatiron (United States) cohorts.

	NICHE—China					Flatiron—United States				
	2003–2007	2008–2012	2013–2017	2018–2023	Total	2003–2007	2008–2012	2013–2017	2018–2023	Total
*N* (%)	64 (3.95)	185 (11.41)	470 (28.98)	903 (55.67)	1622	40 (<1.0)	1357 (10.79)	4999 (39.73)	6186 (49.17)	12,582
Age at diagnosis										
Median (range)	55.5 (37–74)	54.0 (32–83)	56.5 (25–78)	58.0 (25–80)	57.0 (25–83)	61.5 (42–77)	67.0 (23–85)	68.0 (25–85)	69.0 (19–85)	68.0 (19–85)
Age group, *n* (%)										
18–49	19 (29.69)	64 (34.59)	123 (26.17)	174 (19.27)	380 (23.43)	5 (12.50)	109 (8.03)	316 (6.32)	347 (5.61)	777 (6.17)
50–65	37 (57.81)	95 (51.35)	284 (60.43)	528 (58.47)	944 (58.20)	22 (55.00)	495 (36.48)	1783 (35.67)	1955 (31.60)	4255 (33.82)
66–70	5 (7.81)	19 (10.27)	42 (8.94)	127 (14.06)	193 (11.90)	10 (25.00)	216 (15.92)	854 (17.08)	1105 (17.86)	2185 (17.37)
71+	3 (4.69)	7 (3.78)	21 (4.47)	74 (8.19)	105 (6.47)	3 (7.50)	537 (39.57)	2046 (40.93)	2779 (44.92)	5365 (42.64)
Sex, *n* (%)										
Male	43 (67.19)	123 (66.49)	280 (59.57)	499 (55.26)	945 (58.26)	23 (57.50)	716 (52.76)	2706 (54.13)	3357 (54.56)	6820 (54.20)
Female	21 (32.81)	62 (33.51)	190 (40.43)	404 (44.74)	677 (41.74)	17 (42.50)	641 (47.24)	2293 (45.87)	2811 (45.44)	5762 (45.80)
M:F	2.05	1.98	1.47	1.24	1.4	1.35	1.12	1.18	1.20	1.18
ISS stage, *n* (%)										
I	11 (18.97)	39 (21.55)	88 (19.60)	195 (22.47)	333 (21.40)	7 (39.13)	195 (30.36)	901 (33.53)	1261 (33.97)	2364 (33.46)
II	28 (48.28)	73 (40.33)	168 (37.42)	304 (35.02)	573 (36.83)	7 (30.43)	247 (37.76)	894 (33.49)	1226 (32.76)	2374 (33.60)
III	19 (32.76)	69 (38.12)	193 (42.98)	369 (42.51)	650 (41.77)	5 (30.43)	206 (31.87)	879 (32.98)	1238 (33.26)	2328 (32.95)
Missing	6 (9.38)	4 (2.16)	21 (4.47)	35 (3.88)	66 (4.07)	21 (52.50)	709 (52.25)	2325 (46.51)	2461 (39.78)	5516 (43.84)
Myeloma isotype, *n* (%)										
IgG	22 (34.38)	105 (56.76)	222 (47.23)	430 (47.72)	779 (48.36)	31 (77.50)	792 (61.83)	2820 (59.43)	3475 (58.09)	7118 (59.08)
IgA	21 (32.81)	43 (23.24)	106 (22.55)	197 (21.86)	367 (22.78)	6 (15.00)	258 (20.14)	965 (20.34)	1241 (20.75)	2470 (20.50)
Light chain	14 (21.88)	27 (14.59)	97 (20.64)	196 (21.75)	329 (20.42)	3 (7.50)	217 (16.94)	901 (18.99)	1200 (20.06)	2321 (19.26)
IgD	2 (3.13)	7 (3.78)	35 (7.45)	53 (5.88)	95 (5.90)	0 (0.00)	3 (0.23)	20 (0.42)	25 (0.42)	48 (0.40)
IgM	0 (0.00)	0 (0.00)	1 (0.21)	6 (0.67)	7 (0.43)	0 (0.00)	10 (0.78)	35 (0.74)	39 (0.65)	84 (0.70)
Other	5 (7.81)	3 (1.62)	9 (1.91)	19 (2.11)	34 (2.11)	0 (0.00)	1 (0.08)	4 (0.08)	2 (0.03)	7 (0.06)
Missing	0 (0.00)	0 (0.00)	0 (0.00)	2 (0.50)	11 (1.62)	0 (0.00)	76 (5.60)	254 (5.08)	204 (3.30)	534 (4.24)
Cytogenetic risk at diagnosis, *n* (%)										
Standard	13 (65.00)	119 (73.91)	339 (74.67)	625 (77.26)	1096 (75.90)	-	311 (73.87)	1748 (76.27)	2557 (74.16)	4616 (74.92)
High *	7 (35.00)	185 (11.41)	115 (25.33)	184 (22.74)	348 (24.10)	-	110 (26.13)	544 (23.73)	891 (25.84)	1545 (25.08)
Missing	44 (68.75)	24 (12.97)	16 (3.40)	94 (10.41)	178 (10.97)	40 (100.00)	936 (68.98)	2707 (54.15)	2738 (44.26)	6421 (51.03)
Duration of follow-up										
Median (IQR)	52.53 (28.33, 105.45)	56.30 (26.42, 92.45)	62.97 (34.83, 82.45)	28.90 (19, 42.43)	35.64 (22.29, 59.08)	183.30 (142.97, 226.20)	63.77 (26.87, 121.27)	55.20 (20.77, 91.03)	30.07 (15.57, 49.37)	38.83 (17.80, 69.53)

ISS: International staging system, IQR: interquartile range, M:F: male to female ratio, N: number of patients. * High risk with del(17p), t(4;14); t(14,16).

**Table 2:** Demographic and clinical features of patients aged ≤65 years of age in the NICHE (China) and Flatiron (United States) cohorts.

	NICHE—China					Flatiron—United States				
	2003–2007	2008–2012	2013–2017	2018–2023	Total	2003–2007	2008–2012	2013–2017	2018–2023	Total
N	56	159	407	702	1324	27	604	2099	2302	5032
Age at diagnosis										
Mean (SD)	53.4 (7.6)	52 (7.8)	53.7 (7.7)	54.3 (7.4)	53.78776 (7.5)	55.96 (6.5)	55.73 (7.5)	56.64 (7.1)	56.82 (6.9)	56.61 (7.0)
Sex, *n* (%)										
Male	37 (66.1)	105 (66)	241 (59.2)	392 (55.8)	775 (58.5)	18 (66.7)	328 (54.3)	1168 (54.6)	1279 (55.6)	2793 (55.5)
Female	19 (33.9)	54 (34)	166 (40.8)	310 (44.2)	549 (41.5)	9 (33.3)	276 (45.7)	931 (45.4)	1023 (44.4)	2239 (44.5)
M:F	1.95	1.94	1.45	1.26	1.41	2	1.19	1.25	1.25	1.25
ISS stage, *n* (%)										
I	9 (17.6)	38 (24.5)	76 (19.6)	158 (23.5)	281 (22.2)	5 (38.5)	108 (33.9)	443 (37.5)	611 (42.1)	1167 (39.3)
II	24 (47.1)	62 (40)	140 (36.1)	244 (36.3)	470 (37.1)	5 (38.5)	121 (37.9)	391 (33.0)	410 (28.3)	927 (31.3)
III	18 (35.3)	55 (35.5)	172 (44.3)	271 (40.3)	516 (40.7)	3 (23.0)	90 (28.2)	349 (29.5)	430 (29.6)	872 (29.4)
Missing	5 (8.93)	4 (2.52)	19 (4.67)	29 (4.13)	57 (4.31)	14 (51.9)	285 (47.2)	916 (43.6)	851 (37.0)	2066 (41.0)
Myeloma isotype, *n* (%)										
IgG	21 (37.5)	87 (54.7)	187 (45.9)	327 (46.6)	622 (47)	21 (80.8)	339 (60.4)	1176 (59.4)	1294 (59.4)	2830 (59.6)
IgA	15 (26.8)	38 (23.9)	94 (23.1)	148 (21.1)	295 (22.3)	3 (11.5)	107 (119.1)	381 (19.3)	421 (19.3)	912 (19.2)
Light chain	14 (25)	25 (15.7)	83 (20.4)	163 (23.2)	285 (21.5)	2 (7.7)	113 (20.1)	397 (20.1)	442 (20.3)	954 (20.1)
IgD	2 (3.6)	7 (4.4)	34 (8.4)	45 (6.4)	88 (6.6)	0 (0)	1 (0.18)	10 (0.47)	13 (0.51)	24 (0.50)
IgM	0 (0)	0 (0)	0 (0)	0 (0)	0 (0)	0 (0)	0 (0)	12 (0.57)	10 (0.50)	22 (0.46)
Other	4 (7.1)	2 (1.3)	9 (2.2)	19 (2.7)	34 (2.6)	0 (0)	1(0.18)	3 (0.14)	0 (0)	4 (0.08)
Missing	0 (0.00)	0 (0.00)	0 (0.00)	0 (0.00)	0 (0.00)	1 (3.7)	43 (7.1)	120 (5.7)	122 (5.3)	286 (5.7)
Cytogenetic risk at diagnosis, *n* (%)										
Standard	11 (68.8)	101 (74.3)	285 (72.7)	469 (74.8)	866 (74)	-	145 (70.0)	746 (76.3)	934 (73.0)	1825 (74.1)
High *	5 (31.3)	35 (25.7)	107 (27.3)	158 (25.2)	305 (26)	-	62 (30.0)	232 (23.7)	345 (27.0)	639 (25.9)
Missing	40 (71.43)	23 (14.47)	15 (3.69)	75 (10.68)	153 (11.56)	27 (100.00)	397 (65.7)	1121 (53.4)	1023 (44.4)	2568 (51.0)
Duration of follow-up										
Median (IQR)	51.8 (27.8, 104.0)	56.4 (28.9, 97.3)	63.3 (35.1, 82.9)	14.8 (29.5, 20.1)	38.0 (23.5, 61.3)	202.4 (157.2, 232.0)	90.9 (39.6, 146.4)	75.5 (29.1, 100.5)	33.9 (18.5, 53.2)	48.4 (22.3, 82.5)

ISS: International staging system, IQR: interquartile range, M:F: male to female ratio, N: number of patients, SD: standard deviation. * High risk with del(17p), t(4;14); t(14,16).

**Table 3:** Stratified Cox regression of survival in the NICHE (China) and Flatiron (United States) cohorts.

	NICHE—China								Flatiron—United States			
			Univariate Analysis		Multivariate Analysis				Univariate Analysis		Multivariate Analysis	
Variables	N	Event	HR (95% CI)	*p*-Value	HR (95% CI)	*p*-Value	N	Event	HR (95% CI)	*p*-Value	HR (95% CI)	*p*-Value
PFS												
ISS stage												
Stage 1/2 vs. Stage 3	867	410	0.720 (0.623–0.832)	<0.001	0.703 (0.582–0.849)	<0.003	4724	1607	0.539 (0.501–0.579)	<0.001	0.609 (0.551–0.673)	<0.001
Cytogenetic risk												
Standard vs. High	1083	532	0.776 (0.659–0.915)	0.003	0.754 (0.609–0.934)	0.010	4616	1545	0.629 (0.569–0.696)	<0.001	0.622 (0.559–0.693)	<0.001
Induction regimen												
PIs + IMIDs vs. Cytotoxics/PI/IMIDs-based	633	217	0.691 (0.587–0.813)	<0.001	0.797 (0.643–0.987)	0.037	6315	2315	0.724 (0.686–0.764)	<0.001	0.828 (0.742–0.924)	<0.001
CD38 mAb vs. Cytotoxics/PI/IMIDs-based	173	38	0.591 (0.421–0.828)	0.002	0.442 (0.272–0.718)	0.001	1568	272	0.616 (0.556–0.683)	<0.001	0.709 (0.585–0.858)	<0.001
ASCT												
Yes vs. No	548	215	0.492 (0.420–0.577)	<0.001	0.625 (0.508–0.769)	<0.001	2780	808	0.343 (0.317–0.371)	<0.001	0.427 (0.371–0.490)	<0.001
Maintenance												
Yes vs. No	101	57	0.585 (0.444–0.771)	0.0001	0.695 (0.509–0.948)	0.022	4453	1395	0.621 (0.587–0.656)	<0.001	0.824 (0.736–0.922)	<0.001
OS												
ISS stage												
Stage 1/2 vs. Stage 3	867	185	0.557 (0.454–0.683)	<0.001	0.554 (0.419–0.731)	<0.001	4724	1591	0.539 (0.501–0.579)	<0.001	0.588 (0.532–0.650)	<0.001
Cytogenetic risk												
Standard vs. High	1083	260	0.686 (0.548–0.859)	0.001	0.661 (0.486–0.898)	0.008	4616	1743	0.601 (0.544–0.665)	<0.001	0.587 (0.527–0.653)	<0.001
Induction regimen												
PIs + IMIDs vs. Cytotoxics/PI/IMIDs-based	633	72	0.484 (0.370–0.632)	<0.001	0.576 (0.398–0.833)	0.003	6315	2468	0.723 (0.685–0.764)	<0.001	0.915 (0.819–1.002)	0.115
CD38 mAb vs. Cytotoxics/PI/IMIDs-based	173	14	0.526 (0.293–0.946)	0.032	0.569 (0.262–1.238)	0.155	1568	356	0.759 (0.685–0.842)	<0.001	1.040 (0.856–1.264)	0.691
ASCT												
Yes vs. No	548	73	0.357 (0.277–0.461)	<0.001	0.427 (0.304–0.600)	<0.001	2780	649	0.344 (0.319–0.372)	<0.001	0.442 (0.385–0.508)	<0.001
Maintenance												
Yes vs. No	101	30	0.484 (0.330–0.711)	<0.001	0.572 (0.370–0.884)	0.012	4453	1575	0.633 (0.599–0.670)	<0.001	0.847 (0.757–0.948)	0.004

ISS: International staging system, ASCT: Autologous stem-cell transplant.

## Data Availability

The data for this study were obtained under license. Interested researchers may request access to the data through formal application to NICHE from corresponding authors.

## Supplementary Materials

The following supporting information can be downloaded at: https://www.mdpi.com/article/10.3390/cancers18010053/s1, Table S1. Age standardised death rate of the NICHE (China) and Flatiron (United States) cohorts and patients aged ≤65 years of age in the NICHE (China) and Flatiron (United States) cohorts. Figure S1. Study cohort selection. Figure S2. Trends in the use of ASCT (A–B) and survival of ≤65 years old patients who underwent ASCT in the NICHE (C,D) and Flatiron (E–F) cohorts. Figure S3. Maintenance treatment trends in NICHE cohorts (A–D) and Flatiron cohorts (E–H).
