# Supplementary material for: Two Decades of Real-World Study in Newly Diagnosed Multiple Myeloma: Evolving Treatment and Outcomes in China with Reference to the United States
Source: Cancers (Basel). 2025 Dec 24;18(1):53. doi: 10.3390/cancers18010053 (PMC12785032; doi:10.3390/cancers18010053)

Two decades of real-world study in newly diagnosed multiple myeloma: evolving treatment and outcomes in China with reference to the United States

Jingyu Xu, Meng Shu, Hsingwen Chung, Jian Cui, Yuntong Liu, Wenqiang Yan, Qirui Bai, Ning Dai, Lingna Li, Jieqiong Zhou, Yating Li, Chenxing Du, Shuhui Deng, Weiwei Sui, Yan Xu, Hong Qiu, Lugui Qiu, Gang An

SUPPLEMENTARY MATERIAL

Table S1 Age standardised death rate of the NICHE (China) and Flatiron (United States) cohorts and patients aged ≤65 years of age in the NICHE (China) and Flatiron (United States) cohorts

	Standard population	Deaths in standard population	Death rate in standard population	Study cohort	Deaths in study cohort	Death rate in study cohort	Age-standardised death rate
**NICHE – China**							
**Patients**	1,408,350,446	7,965,772	0.5656%	1558	392	25.16%	16.48%
Patients aged≤65	1,222,624,610	1,989,025	0.1627%	1268	311	24.53%	14.58%
**Flatiron – United States**							
**Patients**	340,161,441.00	2,585,519	0.7601%	12582	5509	43.78%	21.99%
Patients aged≤65	284,124,027.50	675,185	0.2376%	5032	1630	32.39%	16.07%

Figure S1 Study cohort selection

MM: Multiple myeloma, N: number of patients, NICHE: National Longitudinal Cohort of Hematological Disease in China
Flatiron patients were selected using simple random sampling.

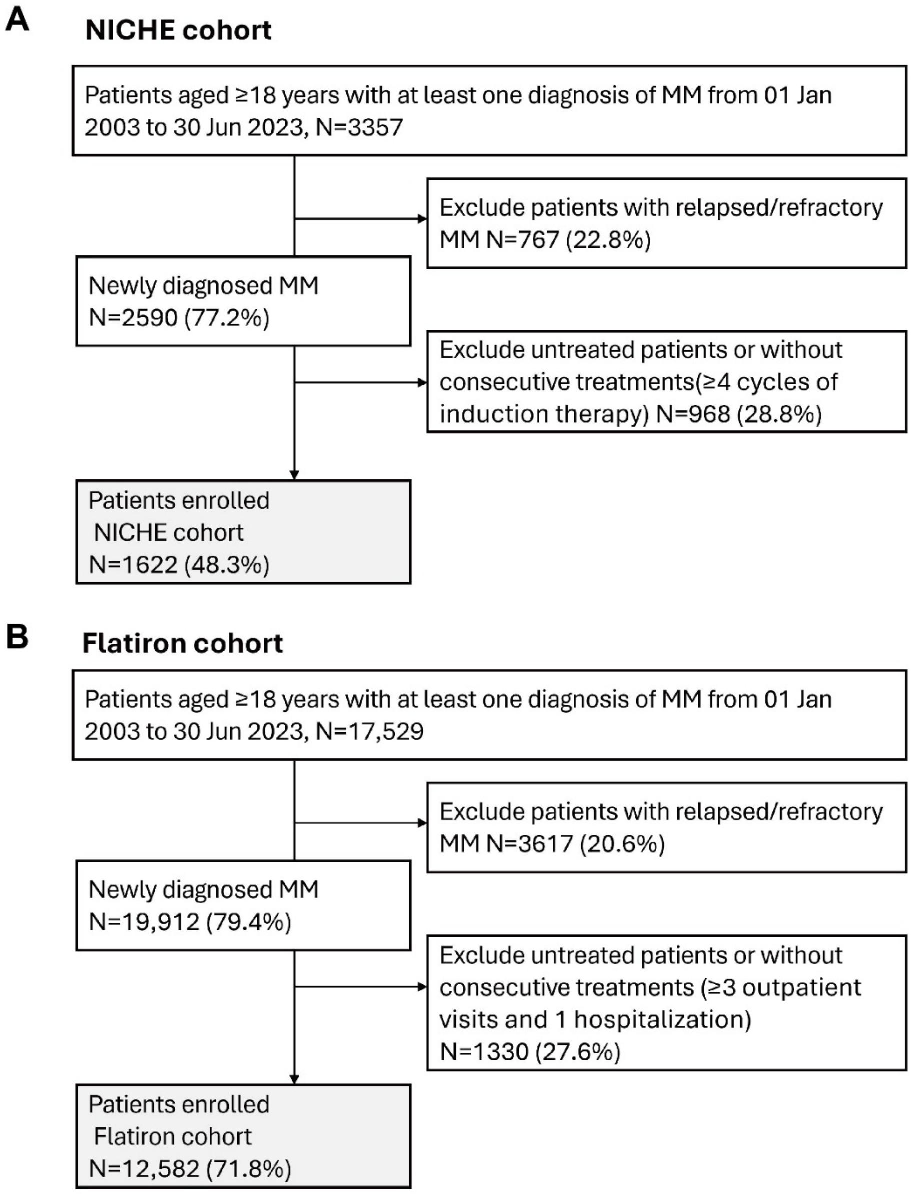

Figure S2 Trends in the use of ASCT (A-B) and survival of ≤65 years old patients who underwent ASCT in the NICHE (C-D) and Flatiron (E-F) cohorts

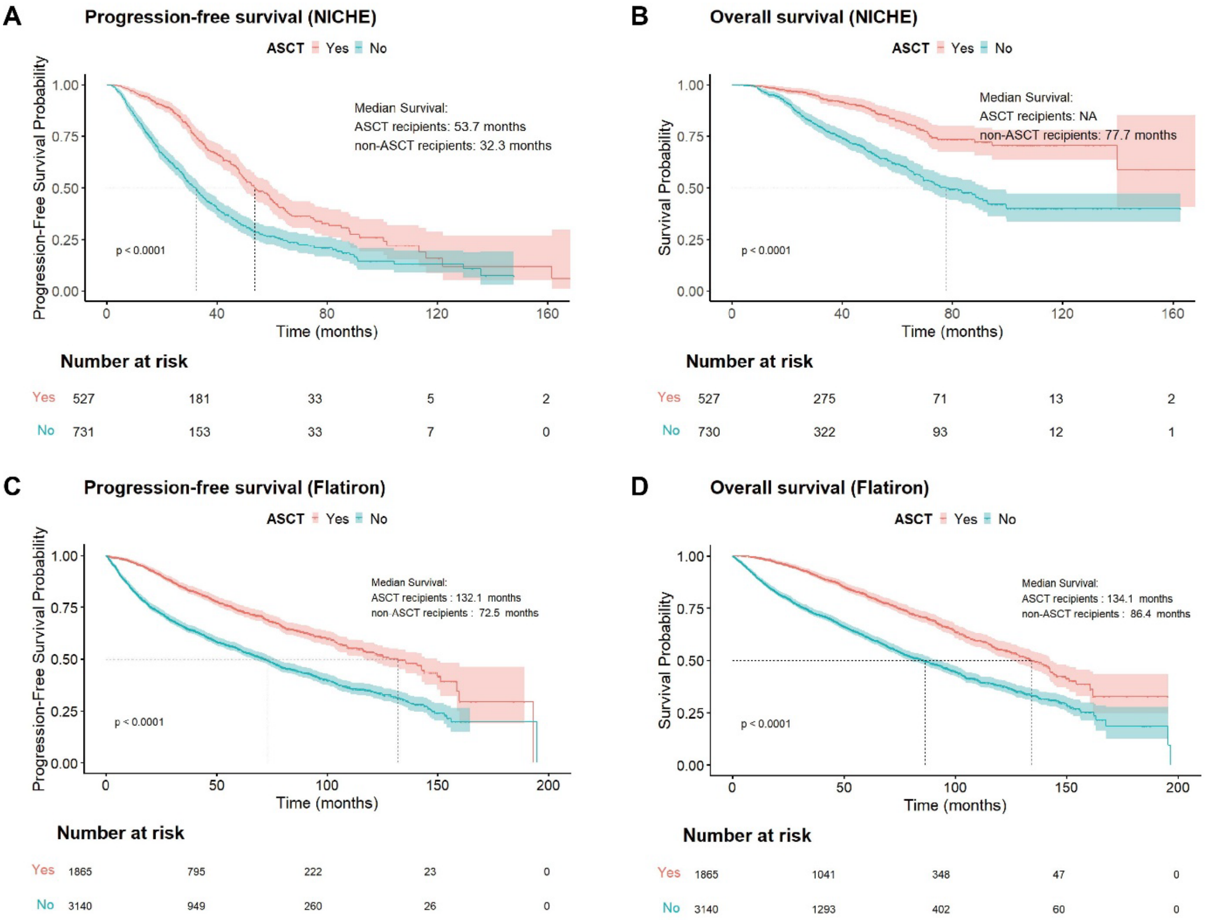

Figure S3 Maintenance treatment trends in NICHE cohorts (A-D) and Flatiron cohorts (E-H)

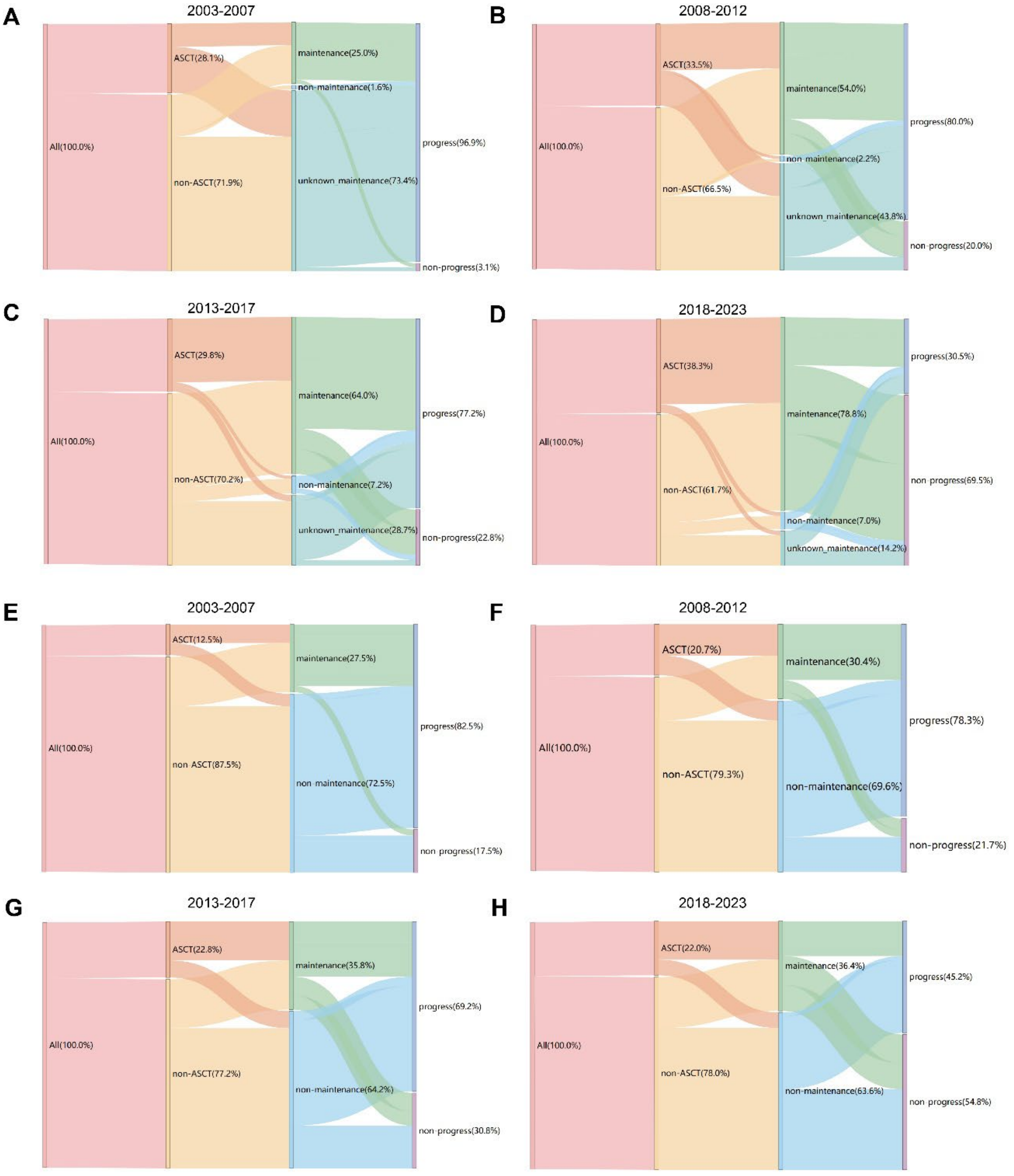

Supplement: Supplementary file 1 [file cancers-18-00053-s001.zip › cancers-3973498-supplementary.pdf]
